# Medicaid Adult Dental Benefit Impact on Dental Utilization: A University Clinic Setting

**DOI:** 10.3389/fpubh.2017.00147

**Published:** 2017-07-04

**Authors:** Lynn Doan, Tamanna Tiwari, Diane Brunson, Clifton M. Carey

**Affiliations:** ^1^University of Colorado School of Dental Medicine, Aurora, CO, United States; ^2^Department of Applied Dentistry, University of Colorado School of Dental Medicine, Aurora, CO, United States; ^3^Department of Craniofacial Biology, University of Colorado School of Dental Medicine, Aurora, CO, United States

**Keywords:** adult Medicaid dental benefit, services, utilization, dental extraction, tooth saving procedures, University Dental School, Colorado

## Abstract

**Introduction:**

In 2014, the state of Colorado initiated new dental coverage benefits for adults in the Colorado Medicaid program. The goal of this study was to investigate the utilization and impact of this new dental coverage at the University of Colorado School of Dental Medicine. The utilization of dental services delivered and the numbers of patients in this program were compared before and after the implementation of the benefit.

**Materials and methods:**

This retrospective study compared the utilization of services provided 2 years prior and 2 years after the Medicaid adult benefit was made available. Through the University of Colorado School of Dental Medicine (CU-SODM) electronic dental record, all adult Medicaid dental patients’ (ages 21+) charts were extracted for zip code, CDT dental procedure codes, with a focus on tooth extraction compared to tooth saving procedures. Graphical analysis and Pearson’s chi-squared tests were applied to assess the statistical significance of procedure utilization changes over time.

**Results:**

After implementation of the Medicaid adult benefit, the number of patients seen at the school under this program increased by a factor of 4.5. The geographic range (zip code) increased with some patients coming from further distances to receive dental care. The number of patients from local zip codes increased by as much as 235%. There was a 51% increase in tooth saving procedures, which was statistically significant (*P* = 0.0013). Additionally, there was a 22% decrease in extractions, while not statistically significant (*P* = 0.0992), a downward trend was clear.

**Discussion:**

The focus was on the utilization of Medicaid adult benefits at the dental school, which was only a small proportion of the state-wide Medicaid population. Therefore, these data are not generalizable for statewide assessments of the program. However, based on the findings at the school clinics, more adult patients utilized the benefits; and chose to receive more tooth saving procedures and less extractions after implementation of the Medicaid adult benefit. This Medicaid study conducted at the CU-SODM 2 years after the adult dental coverage can be used as a baseline for future studies.

## Introduction

The Medicaid dental benefit is one of the few options for low-income adults seeking oral healthcare. Unfortunately, states are inconsistent in what benefits are provided for adult patients. Forty-seven states offer some benefits, but only 15 states offer comprehensive oral care. The adult dental benefits vary greatly between the other states with no minimum requirements for adult dental coverage mandated by the federal government. In addition, the Medicaid benefits are rapidly changing as a function of the economy and regulations. This makes it difficult for many patients to pursue consistent dental care (1, 2).

Several studies have found that without the Medicaid dental benefits, adults are left to seek care in the emergency department (ED) (3). In California, the elimination of dental benefits for Medicaid adult enrollees led to an immediate and significant increase in dental ED visits by Medicaid-enrolled adults (4). Similarly, the elimination of adult Medicaid dental benefits in Oregon in 2003 resulted in an increase in dental-related ED use as well as an increase in the incidence of unmet oral health needs among adult beneficiaries (5).

Conversely, a national analysis found that providing dental benefits may: (1) increase a patient’s likelihood of visiting a dentist within the past 6 months; (2) reduce the likelihood of patients reporting dental needs not being met due to costs; and (3) reduce the likelihood of negative oral health outcomes (6). The advent of the Colorado Medicaid adult coverage in 2014 may have beneficial outcomes that need to be studied.

In 2014, Colorado decided to expand Medicaid under The Patient Protection and Affordable Care Act. Additionally, Colorado added a Medicaid Adult dental benefit for adults ages 21 and over. This new dental benefit provides eligible Medicaid members up to $1,000 in comprehensive dental services per fiscal year. The new benefit covers basic preventative dental exams, diagnostic and restorative dental services, extractions, root canals, crowns, partial dentures, complete dentures, periodontal scaling, root planning, and other procedures (7). Previously, Colorado adults received Medicaid coverage for only emergency dental conditions. Now that Medicaid in Colorado includes this limited adult dental benefit there are now hundreds of thousands of adults expected to seek dental benefits for the first time (8). This study focuses on the effect of the expansion of the Medicaid dental benefits for the adult population and not on various important demographic parameters such as race or ethnicity because the Medicaid expansion was solely based on age. The impact of the new adult Medicaid dental benefit in Colorado is a topic of interest.

Prior research in Colorado compared dental services utilization among the Medicaid population in the state before and after adding the new dental benefit to Medicaid plans. The study found that use of dental services increased after health reform (unpublished study). However, the study was limited to 1 year before and after adding dental benefits. Because the adult dental benefit was new, there were expected challenges in benefit design and administration, notifying people of the new benefit, and recruiting providers.

The current study builds upon the previous research and examines the impact of the Colorado Medicaid policy changes over the period of time 2 years prior through 2 years after the addition of the Medicaid adult dental benefit. Further, this study will focus on the population seen at the University of Colorado School of Dental Medicine (CU-SODM), which no other study has done before.

Our research question was “In the Medicaid adult population seen at the University of Colorado, School of Dental Medicine, what is the effect of the new Medicaid adult benefit on the numbers of patients and types of services utilized, compared before and after the implementation of the benefit?”

## Materials and Methods

This was a retrospective study where patient electronic dental records were examined for patient demographics, zip codes, and dental procedures delivered during the fiscal years (FYs) 2 years before and after the dental benefit. FY2013 and FY2014 are defined as pre-benefit and FY2015 and FY2016 are defined as post-benefit. This study was approved by the Colorado Multiple Institutional Review Board (COMIRB) #16-1156 as Not Human Research.

Through the University of Colorado School of Dental Medicine (CU-SODM) electronic dental record, all adult Medicaid dental patients’ (ages 21+) charts were extracted for CDT dental procedure codes, with a focus on tooth extraction compared to tooth saving procedures.

Reporting of numbers of patients who live in specific zip codes was restricted to those who had a population of 20 or greater who came to the *School* for dental care. Those zip codes where numbers of patients were between 1 and 19 who came to the *School* for dental care were reported with an asterisk. This was done to assure patient anonymity.

Table 1 includes all the procedure codes used in this study. For the purpose of our study, restorative procedures, periodontal treatment, and endodontic treatment were categorized together as tooth saving procedures. The percentage of received dental services was normalized by dividing the number of specific procedures by the total number of procedures completed in that fiscal year.

**Table 1:** CDT procedure codes used in this study.

Prevention	Periodontal	Fluoride treatment	Restorative	Endodontics	Pros-complete	Pros-partial	Bridges	Extractions
1,110	4,341	1,206	2,140–2,394	3,310–3,330	5,110	5,211–5,214	6,211–6,791	7,140
	4,342				5,120	5,221–5,224		7,210–7,250
	4,355				5,130	5,281		
	4,910				5,140	5,820		
					5,810	5,821		
					5,811			

Graphical analysis and Pearson’s chi-squared tests of the percent of received procedures were applied to assess statistical significance over time (9). These data are categorical; therefore, we did not average the number of procedures received. Pearson’s chi-squared tests were used to evaluate the statistical significance of the changes in procedure utilization over the 4-year period of our study. The statistical analyses were performed using each year of the study to assess significance of the changes year to year. FY2013 was compared to FY2016 and FY2014 was compared to FY2015. This approach evaluates both the end points and the interim changes over the 4 years of our study. Additionally, zip code mapping was conducted to evaluate any changes in the service area of where the patients live over the years.

## Results

### Number of Adult Medicaid Patients Seen by Fiscal Year

The total number of adult Medicaid patients seen at the CU-SODM increased from 632 in FY2013 to 2,843 in FY2016, an increase of almost 350%, from FY2013 to FY2016. The population growth in the 26 zip codes where the greatest number of patients live increased from 954,953 in FY2013 to 1,012,334 in FY2016, an increase of 6.0% [data extracted from Ref. (10, 11)].

**Figure 1:**
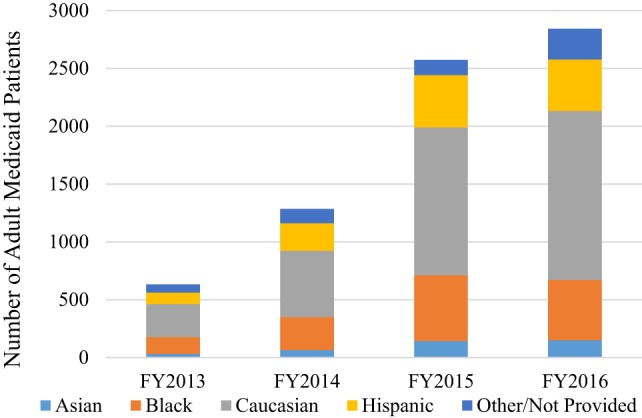
Number of adult Medicaid patients seen and demographic breakdown by Fiscal Year 2013–2016.

The relative percentage of the demographics of the adult Medicaid patients remained consistent pre-benefit and post-benefit, with the majority of patients being women and non-Hispanic (Figure 1). This is also reflective of the overall Medicaid population in the *School’s* traditional service area within the state. The numbers of individuals seeking dental care in this program increased for all of the demographic categories. Additionally, the racial make-up of the study population also remains consistent pre- and post-benefit, with the majority of patients being non-Hispanic white (Figure 1).

### Geographic Profile by Zip Code

**Figure 2:**
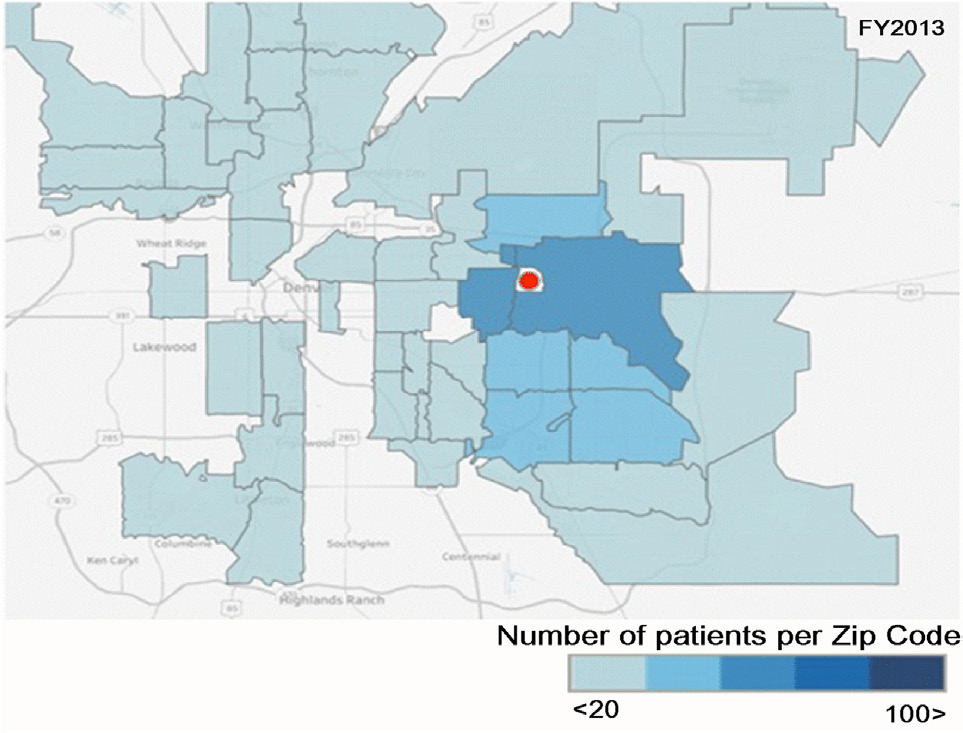
The number of Medicaid adult patients by zip code for FY2013. Red circle is the location of the CU-SODM.

**Figure 3:**
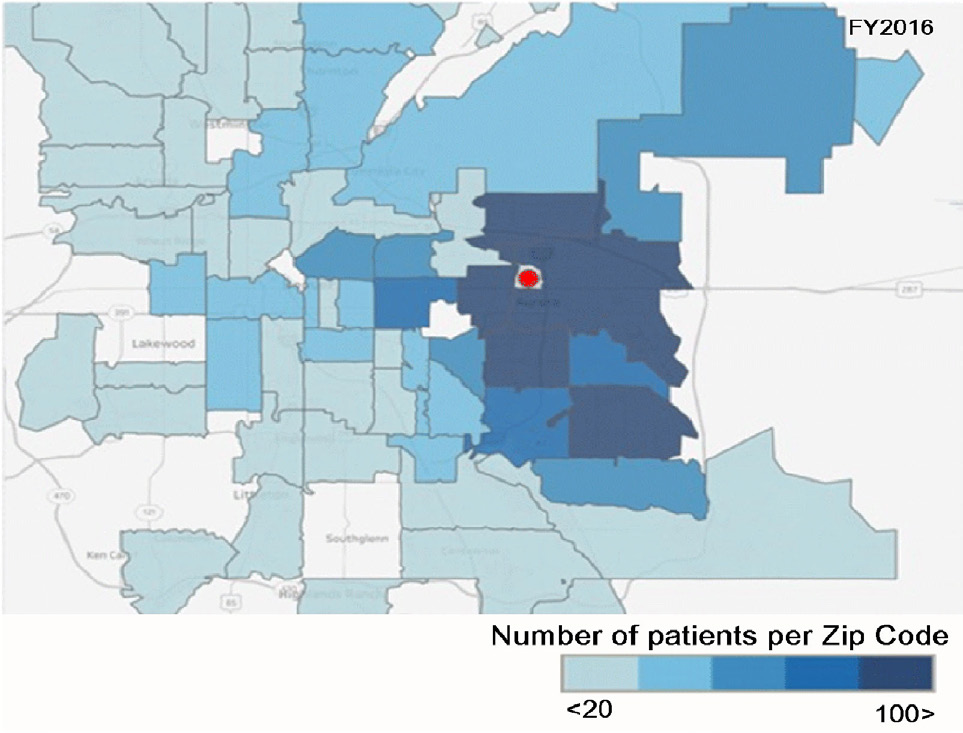
The number of Medicaid adult patients by zip code for FY2016. Red circle is the location of the CU-SODM.

Over the four FYs, the geographic service range increased with a larger number of patients coming from zip codes that are further away to receive dental care. The biggest increases occurred in zip codes that were closer to the school. Additionally, access to care was also expanded within our community. There was an increase from 91 patients in FY13 to 295 patients in FY16, a 3.3-fold increase, which came from the two zip codes immediately surrounding the *School* (80011 and 80010) with similar increases in patients from adjacent zip codes as shown in Figures 2 and 3. The number of Medicaid enrolled adults by zip code is not available for Colorado. However, if one presumes that the number of Medicaid enrolled adults in the two counties that include these two zip codes is indicative, then a perspective of the impact of the expansion of the Medicaid benefits enacted for FY2013 may be obtained. The number of Medicaid enrolled adults increased dramatically increased from 24,689 (FY2013) to 62,676 (FY2016) in Adams County (zip code 80011) and 25,839 (FY2013) to 66,382 (FY2016) in Arapahoe County (zip code 80010). The growth in enrolled adults into Medicaid increased by a factor of 2.5 from FY2013 to FY2016 [data extracted from Ref. (12)].

### Utilization of Services

**Figure 4:**
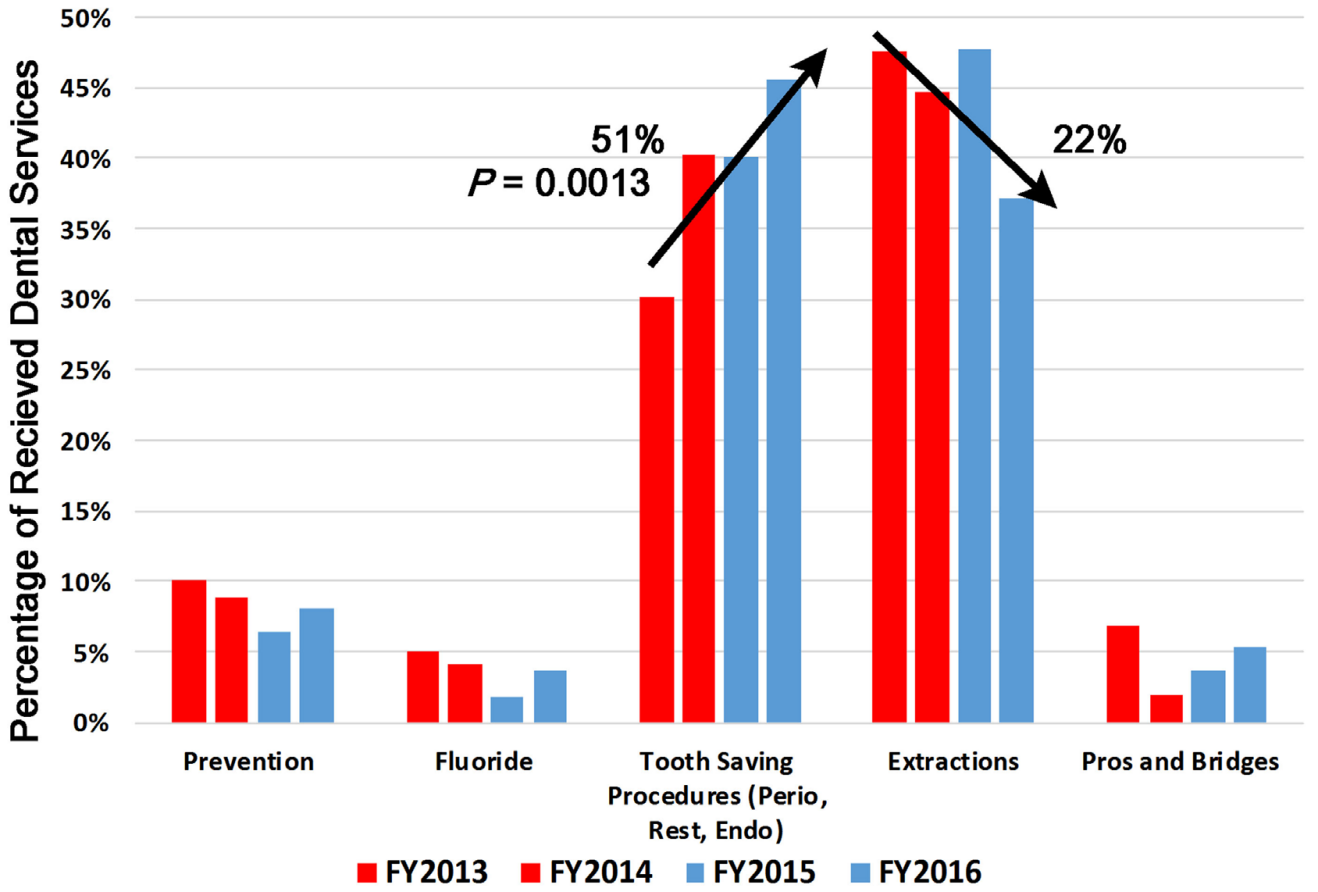
Percentage of received dental services by procedure type for FY2013–2016.

**Figure 5:**
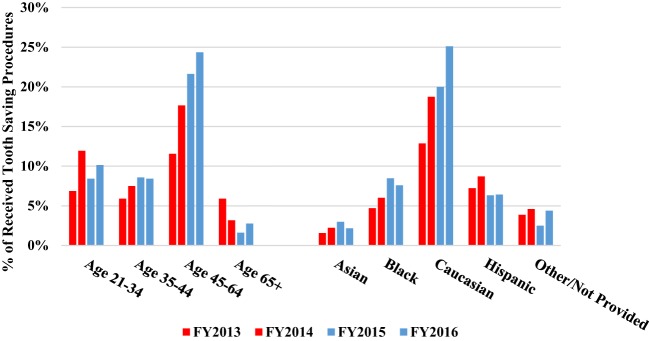
Demographics of tooth saving procedures by age groups and ethnicity for FY2013–2016.

**Figure 6:**
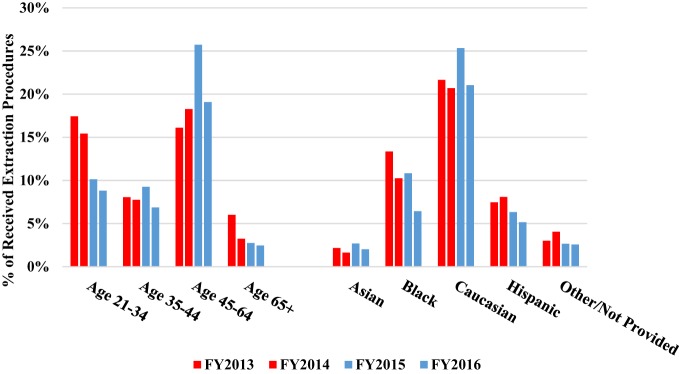
Demographics of extractions by age groups and ethnicity for FY2013–2016.

In this study, over the period from pre-benefit to post-benefit, there was an increase in utilization upon the availability of the benefits as shown in Table 2. Pearson’s chi-squared tests were utilized to assess significance in the change of tooth saving procedures and tooth extraction procedures over time. Tables 3 and 4 show these chi-squared tests. There was a 51% increase in tooth saving procedures delivered, which was statistically significant (*P* = 0.0013). Additionally, there was a 22% decrease in extraction procedures performed, while not statistically significant (*P* = 0.0992), the downward trend was clear as shown in Figure 4. The percent of tooth saving procedures increased in all age groups and ethnicities (Figure 5). The percent of extraction procedures decreased in all, but the 45–65 age groups (Figure 6).

**Table 2:** Proportion of procedures and total number of procedures delivered by year.

Procedure delivered	FY2013	FY2014	FY2015	FY2016
Prevention (%)	10.22	8.96	6.42	8.19
Fluoride (%)	5.05	4.11	1.81	3.64
Tooth saving procedures (Perio, Rest, Endo) (%)	30.17	40.23	40.22	45.63
Extractions (%)	47.60	44.68	47.84	37.20
Pros and bridges (%)	6.97	2.02	3.71	5.34
No. procedures delivered (%)	832	1,484	6,353	7,436

**Table 3:** Pearson’s chi-squared statistical analyses of the change in tooth saving procedures over time: Perio + Restorative + Endo.

FY2013 vs. FY2016			FY2014 vs. FY2015	
Before	30.2		Before	40.2
After	45.6		After	40.2
Change	15.5		Change	0.0

		**Tooth saving**		
**Crosstabulation**		**Before**	**After**	**Total**

FY2013 vs. FY2016	Count	30.2	45.6	75.8
	Exp count	43.0	32.8	75.8
	Chi^2^	3.840	5.041	8.88
	Std. Resid	−1.960	2.245	
FY2014 vs. FY2015	Count	40.2	40.2	80.5
	Exp count	45.7	34.8	80.5
	Chi^2^	0.647	0.849	1.50
	Std. Resid	−0.804	0.922	
Total count	Count	70.4	85.9	156.3
	Exp count	88.7	67.6	156.3
	% within category	45.1	54.9	100.0
	Chi^2^	10.377		
	Deg freedom	1		
	*P*	0.00127565		

**Table 4:** Pearson’s chi-squared statistical analyses of the change in tooth extraction procedures over time: ADA codes 7140 + 7210 + 7250.

FY2013 vs. FY2016			FY2014 vs. FY2015	
Before	47.6		Before	44.68
After	37.2		After	47.84
Change	−10.4		Change	3.16

		**Extractions**		
**Crosstabulation**		**Before**	**After**	**Total**

FY2013 vs. FY2016	Count	47.6	37.2	84.8
	Exp count	48.1	36.7	84.8
	Chi^2^	0.006	0.008	0.01
	Std. Resid	−0.077	0.088	
FY2014 vs. FY2015	Count	44.68	47.84	92.52
	Exp count	52.5	40.0	92.52
	Chi^2^	1.169	1.535	2.70
	Std. Resid	−1.081	1.239	
Total count	Count	92.28	85.04	177.32
	Exp count	100.7	76.7	177.32
	% within category	52.0	48.0	100.0
	Chi^2^	2.718		
	Deg freedom	1		
	*P*	0.09920717		

## Discussion

The aim of this study was to compare dental services utilization at the University of Colorado School of Dental Medicine (CU-SODM) 2 years before and after the advent of the new adult Medicaid dental benefit to Medicaid plans, with a focus on tooth saving procedures vs. extractions. The COMIRB restriction to protect patient privacy was that this study would not provide the number of patients from any zip code where less than 20 patients came to the *University* for dental care resulted in patient counts from 26 zip codes. There was a dramatic increase in the number of adult Medicaid patients seen in the CU-SODM dental clinics over the 2 years since the inception of the adult Medicaid benefit (Figure 1). The 350% increase in the number of Medicaid patients seen at the *University* is far greater than the 6.0% increase in the general population of the 26 zip codes over the same period of time. This shows that the expansion of the adult Medicaid dental benefit in Colorado is being utilized by a larger percentage of the population than prior to the implementation. This is consistent with similar observations made in states such as Massachusetts when they started their expanded dental care program (13) as well as Iowa and Washington states where there has been a large increase in adults receiving a dental service since the inception of their respective adult Medicaid based programs (14).

In general, the most frequent two procedures received as the CU-SODM were tooth saving procedures and extractions, compared to preventative procedures, fluoride treatment and prosthodontic procedures (Figure 4; Table 2). Compared to the pre-benefit period, patients chose to receive more tooth saving procedures and less extractions. Patients in all but the 45–65 age groups reduced the amount of extraction procedures (Figure 5). This finding could be due to pent-up demand in patients of that age group who may have delayed any dental treatment until the adult Medicaid benefit became available to them.

Our findings show early signs of adult patients making choices to save their teeth upon availability of the adult Medicaid benefits. These findings can be linked to the fact that there are differences in dental utilization based on insurance types (15). For instance, tooth extractions are the most likely option for the low-income population with no or emergency-only benefits. A comparative study in Iowa reported that those with public insurance were four times more likely to have had a tooth extracted than those with private insurance (16). However, multiple studies show that improved oral health is associated with a decrease in chronic disease risk; and tooth retention in particular may extend lifespan (17, 18). A recent critical review found evidence that retention of teeth is associated with better oral health-related quality of life (19). Furthermore, this study highlights the importance of the Medicaid adult dental benefit on various types of dental treatments that may predict better overall health outcomes and quality of life for patients.

This is the first adult dental Medicaid study conducted at the CU-SODM and can be used as a baseline for future studies. It is important to acknowledge that our data are not readily generalizable to the state-wide adult Medicaid population. Compared to the state, the dental school clinics provided services to a small proportion of this population. Additionally, the university clinic setting is a unique teaching environment and is different than what is seen at general health-care clinics. For example, there are differences in the types of procedures and treatment planning, and patients are not seen over a long period of time.

Given the importance of health-care reform and the changes to Medicaid benefits on a frequent and irregular basis, more studies are needed to track how the Medicaid program is improving oral health. This study focused on tooth saving vs. tooth extraction choices made by adults and their caregivers in this study. Further studies should evaluate the impact of the availability of adult Medicaid dental benefits on other important demographic classes such race, gender to ethnicity to assess the penetration of the program to underserved populations around the *Dental School*. Our study will be shared with stakeholders throughout the state including Colorado’s Medicaid Program—Health First Colorado—and other dental schools around the nation to encourage a statewide and national analysis done following our methods.

Because oral health is a critical component of general health, future research should evaluate individual-level data and analyze the impact of dental benefits on other health outcomes such as chronic diseases.

## Conclusion

Our findings at the University of Colorado School of Dental Medicine provide evidence that offering adult Medicaid dental benefits can improve dental care access and use of comprehensive services. This study highlights the importance of the Medicaid adult dental benefit on various types of dental treatments that may predict better overall health outcomes and quality of life for patients. The adult Medicaid benefit, dental school, and dental students are improving the lives of our community around us.

## Author Contributions

LD: study design, data evaluation, writer. TT: data evaluation, writer. DB: study design, data evaluation, writer. CC: study design, data evaluation, writer.

## Conflict of Interest Statement

The authors declare that the research was conducted in the absence of any commercial or financial relationships that could be construed as a potential conflict of interest.
